# 

**Published:** 1913-08-02

**Authors:** 


### Gifts and Bequests.

Lord Avebury has bequeathed £500 to the Royal Maternity Charity of London and £500 to the Royal London Ophthalmic Hospital. He also left £100 to his nurse.

Lord Peckover, of Wisbech, who has already given most generously towards the funds of Addenbrooke's Hospital, Cambridge, in years past, has given £250 towards the cost of providing the new electric lift. It will be remembered that the hospital is entirely indebted to Lord Peckover for the present nurses' home and the operating theatre.

### LEWIS'S POCKET CASE-BOOK.

We have received from Mr. H. K. Lewis a new *Pocket Case-Book* designed for the use of students and practitioners. The book is neatly bound in limp cloth, and the page measures 8 in. by 5 in. It is arranged for twenty-five cases; four pages are allotted to each case, and the headings are arranged for the record of the usual particulars, including personal history, family history, and present condition. The space allotted to the family history is, we think, unnecessarily large compared with that available for personal history. Otherwise the arrangement of the whole case-book is admirable. There are also diagrams for the marking of physical signs, space for diagnosis, prognosis, and extra space for the record of treatment and progress, including a miniature temperature chart which should be very useful. The price is 1s. 6d. net.

